# Unveiling the Dengue Knowledge Gap and Symptom Awareness Among Students of Shahjalal University of Science and Technology, Sylhet: A Cross‐Sectional Study

**DOI:** 10.1002/hsr2.72208

**Published:** 2026-03-26

**Authors:** Md. Fakrul Islam, Md. Efty Islam Arpon, Md. Nazmul Alam, Md. Abu Tawab Hridoy, Fahmida Afroze, Md. Mazharul Islam, Md. Azizul Baten

**Affiliations:** ^1^ Department of Statistics Shahjalal University of Science and Technology (SUST) Sylhet Bangladesh

**Keywords:** attitude, dengue fever, KAP, knowledge, practice, SUST, Sylhet, university students

## Abstract

**Background and Aims:**

Dengue, a significant global public health concern, effectively managing the spread of dengue requires a comprehensive understanding of the knowledge, attitudes, and practices (KAP) of the general community toward dengue prevention. The objective of this study is to assess the knowledge, attitudes, and practices regarding dengue fever among Shahjalal University of Science and Technology students, with a specific focus on identifying gaps in symptom awareness.

**Methods:**

This cross‐sectional study was conducted at Shahjalal University of Science and Technology (SUST), Sylhet, Bangladesh. All six schools of SUST were included, and a stratified random sampling approach with proportional allocation was used to select 419 students. Data were collected using a structured questionnaire adapted from a previously published study and validated by experts. Descriptive statistics, Kolmogorov–Smirnov tests, and Spearman's rank correlation were used to analyze knowledge, attitudes, and practices (KAP) related to dengue. Statistical analyses were performed using SPSS, with a significance level set at 0.05.

**Results:**

This study was conducted based on 419 participants and the average age of the participants was 23.14 ± 0.96 years. Of them, 37.7% were women and 62.3% were men. Twenty three point six percent had previously experienced dengue, either personally or in their family, but the other 76.4% were non‐experienced. The data show that respondents' levels of good knowledge (68.3%), good attitude (85.9%), and good practice (75.9%) are generally high. Most recognized symptoms were Fever (98.8%), joint pain (94.3%), and restlessness (74.0%). Awareness was highest for fever (98.8%), joint pain (94.3%), and headache (84%). In contrast, fewer participants recognized tree branches (36.5%) and flowing water (21.2%) as potential breeding sites. Only 58.7% were aware of medicines like paracetamol or anti‐allergic drugs, and 30.5% knew of herbal remedies. Weak correlations were seen between knowledge and practices (*r* = 0.053, *p* = 0.278) and knowledge and attitudes (*r* = 0.025, *p* = 0.612) after the analysis. There was a negatively strong but not statistically significant relationship between attitudes and practices (*r* = −0.092, *p* = 0.061).

**Conclusion:**

Some symptoms, such as cold, swollen glands, stomach ache, back pain, etc., are less noticeable to students. Some pupils lack the knowledge necessary to prevent dengue and are unaware of its breeding grounds. The knowledge behavior gap should be minimized. For dengue education programs to be effective, they should highlight less prevalent symptoms, make use of social media, and encourage proactive preventive measures. Also, participation in community prevention initiatives should be emphasized.

**Abbreviations**
A.Score: attitude score
DF: dengue fever
KAP: knowledge, attitude and practice
K.Score: knowledge score
P.Score: practice score
SUST: Shahjalal University of Science and Technology

## 1 Introduction

Dengue, a virus transmitted by mosquitoes, poses a significant threat to global public health and is rapidly spreading across all regions designated by the World Health Organization (WHO) [1, 2]. Over the past 50 years, dengue has become one of the most serious vector‐borne virus infections in the world due to its 30‐fold increase in incidence [3]. In many Asian and Latin American nations, children are primarily affected by severe dengue, also referred to as dengue hemorrhagic fever, which can lead to serious sickness and even death [4]. In addition to the high expenses of healthcare, dengue has a considerable negative economic impact on society, especially in regard to loss of productivity and financial strain on families [5]. One of the main causes of the increasing frequency and geographic spread of dengue fever is climate change, together with intense urbanization [6]. The total number of dengue cases worldwide increased from 23,283,274 in 1990 to 104,771,911 in 2017 [7].

The rise in dengue incidence presents serious challenges for Bangladesh's healthcare system, underscoring the need for better diagnosis and treatment procedures [8]. In 2000, there was the first documented dengue outbreak, with 5551 cases and 93 recorded fatalities [9]. According to one estimate, there were 2430 dengue cases reported in 2001, 6232 in 2002, 3934 in 2004, 3162 in 2015, 6060 in 2016, and 10,148 in 2018 [10]. The year 2019 marked a critical juncture in the dengue situation in Bangladesh, reporting the highest annual incidence ever recorded [11]. From January 1, 2023, to August 7, 2023, the Ministry of Health and Family Welfare in Bangladesh recorded a total of 69,483 confirmed cases of dengue and 327 associated deaths. The case fatality rate (CFR) during this period was 0.47% [11]. The increasing incidence of dengue fever in Bangladesh, especially in the country's heavily populated cities, emphasizes the urgent need for coordinated vector control and public health initiatives [12].

Aedes mosquito is found throughout South Asian nations, which indicates a significant risk of transmission. In many regions of South Asia, urbanization, high population density, and unsanitary circumstances provide mosquito vectors' ideal breeding grounds [13]. According to research, 978 patients (23%) out of the total samples were positive for dengue in India [14]. Laboratory‐confirmed dengue infection prevalence among clinically suspected individuals was estimated to be 38.3% (95% CI: 34.8%–41.8%) [15]. Although there have been reports of endemic dengue in Nepal since 2006, the number of cases has grown with time, with 68 districts reporting a total of 17,992 dengue cases in 2019. In 2018, the case incidence was almost five times higher than in 2016, and in 2019, it was more than 140 times higher [16].

A previous survey found that the DF statistics were practice (25.9%), knowledge (47.9%), and attitudes (80.3%). There was a substantial correlation (*p* < 0.05) between good climate change adaptation or mitigation practices and good knowledge and attitudes. A substantial correlation was also identified between good DF prevention practices and good knowledge, attitudes, and prior DF experiences (*p* < 0.001) [17]. Moreover, a study conducted a knowledge, attitude, and practice (KAP) survey on dengue fever (DF) among university students in Bangladesh. The study revealed that the knowledge, attitudes, and practices of the respondents significantly correlated with their academic attainment [18]. Another study involving 1008 participants previously conducted a similar KAP survey [19]. Most individuals were unaware of the breeding and life cycles of dengue vector mosquitoes, despite recognizing the severity of dengue. According to more than 70% of respondents, all members of the family use insecticide‐treated nets (ITN) at night [20]. Despite continuous advancements in dengue research, ensuring appropriate treatment and prevention options is still a challenge, making effective dengue prevention and control a pressing concern in Bangladesh today [21, 22]. Previous research has demonstrated that social incognizance, poverty, and illiteracy are the causes of inadequate dengue management [4]. For the purpose of reducing dengue fever overall, it is crucial to understand the nation's population's knowledge, attitudes, and habits related to the disease. This article focuses on these aspects.

The current vaccine does not provide sufficient protection against all four types of dengue virus, and the current treatment for dengue focuses solely on managing symptoms [21]. Consequently, the main preventive measures involved controlling mosquito vectors and minimizing human‐vector contact [22]. An effective primary school‐based program that engaged children in dengue prevention significantly reduced mosquito larval indices in both students' homes and primary schools [23].

Following a first outbreak in 2000 that resulted in 5551 cases and 93 fatalities, DF has become a serious public health concern in Bangladesh [24]. The first evidence of dengue infection in Bangladesh was found during a late‐summer 1964 outbreak of “Dacca Fever,” a feverish disease that struck the country's capital (now Dhaka) [25]. Although they were not formally documented, occasional instances and little outbreaks that were clinically suggestive of dengue occurred all over the nation between 1964 and 1999 [26, 27]. Three major cities (Dhaka, Chittagong, and Khulna) and 17 towns recorded 5551 instances in 2000 [24], with 1.7% of those cases resulting in death. Of these, 4385 (79.0%) cases were DF, and 1166 (21.0%) were DHF [28]. Between January 2000 and December 2014, the Directorate General of Health Services received reports of over 28,000 illnesses and 242 fatalities [24].

Since university students have access to authentic information, they are an important segment of the learning population in every community. They can readily be contacted by the authorities via various digital and social media platforms or through institutions [28, 29]. These students can contribute to their communities if they possess sufficient information and favorable attitudes. Students can therefore serve as a vital center for community development. This study used the KAP survey paradigm to assess the DF status and responses of university students. In the end, our study's findings can help the government, non‐governmental organizations, university administration, and health and social workers promote comprehensive DF preparedness and response for university students, which can help lower the DF outbreak nationwide.

The objective of this study is to investigate the knowledge, attitudes, and practices related to dengue fever among students at Shahjalal University of Science and Technology (SUST), with a particular focus on identifying gaps in their awareness of Dengue symptoms. Since students come from various regions for education and travel across the country, they could spread awareness and preventive measures against dengue fever. Though there are many studies available in this topic [17, 30, 31, 32], no prior studies on SUST students have been investigated to determine their awareness towards dengue. The recent predicament of the dengue pandemic in Bangladesh turned into an alarming situation, which prompted us to research this contemporary topic at SUST.

## 2 Methodology

### 2.1 Study Design and Survey Area

This study employed a quantitative research design to systematically investigate the knowledge, attitudes, and preventive practices related to DF among university students at SUST.

**Figure 1:**
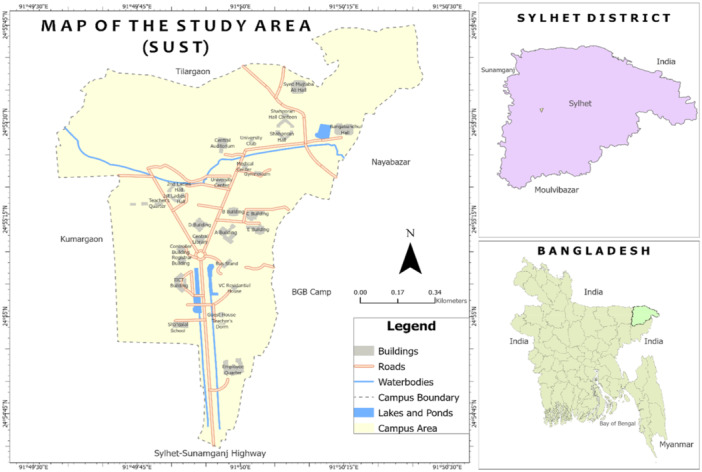
MAP of the study area (SUST).

This design allows for the collection of structured, numerical data, facilitating statistical analysis to uncover patterns, relationships, and associations within the study population. This study adopts a cross‐sectional approach, providing a snapshot of information at a specific point in time. The study was conducted over 5 months, from August 2023 to December 2023 (Figure 1), which was created by the authors. This manuscript was prepared in accordance with the Strengthening the Reporting of Observational Studies in Epidemiology (STROBE) guidelines for cross‐sectional studies, and the completed STROBE checklist is provided in Supporting Information S2.

### 2.2 Questionnaire and Data Collection Procedures

Primary data were collected by a face‐to‐face interview of each individual from each school of different department using a structured questionnaire. The study questionnaire was adapted from a previously published study by researchers at the University Malaysia Sabah (UMS) on KAP related to dengue. It was originally approved by the UMS ethics committee (Ethics reference number [33]: dlm. JKN (SB)100‐13) and complied with the Declaration of Helsinki [34]. To align with the specific objectives of this study, the questionnaire was carefully reviewed and modified as needed. The adapted version retained key domains, including socio‐demographic characteristics, knowledge, attitudes, and practices, ensuring a comprehensive and relevant data collection process. The full questionnaire is provided in the Supporting Information S1.

For data collection, six trained data enumerators were recruited for this study. Prior to data collection, an extensive training workshop was conducted by Prof. Dr. Azizul Haque Baten to enhance data collection efficiency and standardization. Each enumerator was assigned the responsibility of collecting data from 70 participants and was instructed to provide a brief introduction to respondents before administering the questionnaire.

To maintain accuracy and minimize respondent fatigue, each enumerator was restricted to collecting data from only five participants per day. Enumerators ensured that all questions were answered carefully and completely before concluding each interview, ensuring that no missing data occurred in this study. After data collection, the enumerators were responsible for entering the collected data into the study database on a daily basis. Additionally, they generated basic descriptive reports to monitor data consistency and identify any potential discrepancies. The supervisor regularly reviewed the collected data and provided immediate feedback to enumerators to maintain data integrity. Through these rigorous procedures, data quality was continuously assessed and upheld throughout the study.

#### 2.2.1 Score Grading

Among the 18 knowledge‐related questions in the questionnaire, we selected the first 13 key questions for scoring as they comprehensively assess fundamental knowledge of dengue, while the remaining questions provide further elaboration rather than contributing to the total score. The knowledge scores ranged from 0 to 13 points. We used modified Bloom's cutoff points [35] to classify scores into three levels: excellent (85%–100%) for scores between 11 and 13, good (60%–84%) for scores between 8 and 10, and poor (less than 59%) for scores between 0 and 7. The attitude section had 15 statements rated on a Likert scale [36]. Statement scores ranged from strongly agree (5) to strongly disagree (1). Individual scores ranged from 15 to 75, and categorizing them as excellent (60+), good (46–60), and poor attitude (11–45). Practice responses were assessed as zero‐one indicators, with one for “yes” and zero for “no.” The classifications for practice were excellent (8–10 scores), good (6–7 scores), and poor (0–5 scores) based on modified Bloom's cutoff points.

### 2.3 Study Variables

We collected information on students' backgrounds, such as their study year, age, department, family income, and residence type. We also checked whether they or their family members had previously experienced DF. Additionally, we examined their (a) understanding of DF, including symptoms, vectors, management, and prevention; (b) feelings toward DF; and (c) actions taken to prevent and control DF, such as reducing breeding sites and protecting against mosquitoes using methods such as bed nets, repellents, and window screens. Participants were also asked where they received information about DF.

### 2.4 Sampling Technique and Sample Size

**Figure 2:**
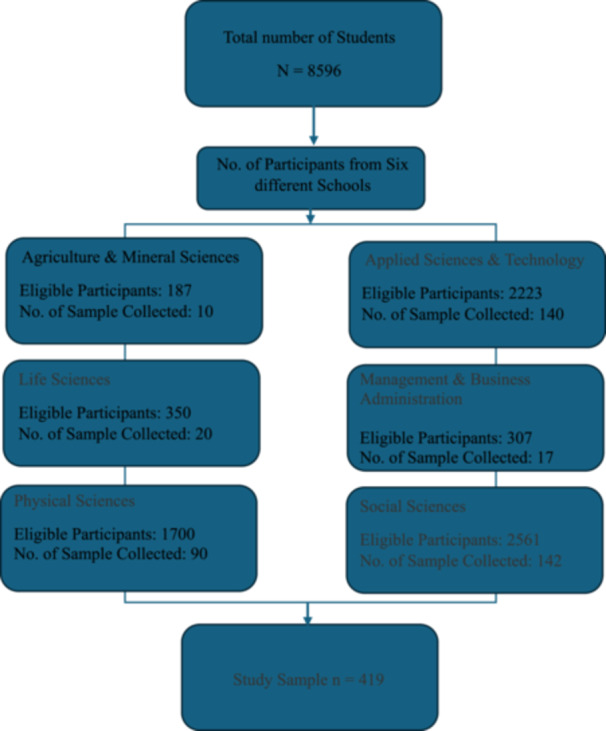
Flow chart of study sample collection. The boxes show the total number of participants who attended from different schools.

The respondents were university students selected through a stratified random sampling method, ensuring representation from six different schools within SUST. We used proportional allocation in the sampling technique to determine the required observations for each school. Then we randomly selected the required sample from each school using simple random sampling. The number of respondents from each school is provided in Figure 2.

The sample selection procedure is as follows:

The sample size was calculated using the following formula:

$$n=z^{2}\left[P\left(1-P\right)/d^{2}\right]$$

As there were no similar studies related to dengue fever, the calculations were based on the assumption that the probability of having good knowledge of and a positive attitude toward preventive measures against dengue fever is the most conservative estimate of the indicator percentage (50%) [37]. At the 95% confidence interval, the limit of precision is 0.047%, the relative precision is 0.047P, the calculated sample size is 419 participants in the SUST for dengue fever, and this is a statistically representative sample.

It is expected that the 419 sample in the SUST will be sufficient for studying any type of indicator because this sample is widely used by the Bangladesh Bureau of Statistics (BBS) and UNICEF for conducting multiple indicator cluster surveys [38].

### 2.5 Statistical Analysis

To investigate participant characteristics and the KAP domain, all statistical analyses were carried out within a single analytical framework. The Shapiro–Wilk and Kolmogorov–Smirnov tests were used to evaluate the normality of the data. The continuous variables were not normally distributed; when necessary, suitable nonparametric techniques were used. Frequencies and percentages were used to summarize categorical factors, such as academic and sociodemographic traits. Means and standard deviations were used to describe continuous data, such as age and income. Because of the data's non‐normal distribution, associations within the KAP domain were evaluated using Spearman's rank correlation coefficient. *p* values less than 0.05 were regarded as statistically significant. Microsoft Excel and SPSS Version 27.0 were used for statistical analysis.

#### 2.5.1 Software and Technical Support

Statistical analysis was conducted using SPSS (IBM Corp. Released 2020. IBM SPSS Statistics for Windows, Version 27.0. Armonk, NY: IBM Corp) and Microsoft Excel. These tools facilitate rigorous analysis and presentation of findings, ensuring accuracy and reliability.

## 3 Results

### 3.1 Results of Socio‐Demographic and Socio‐Economic Characteristics

A total of 419 participants participated in this study with a mean age of 23.14 ± 0.96 years. The 62.3% were male, while 37.7% were female. Among the schools, 33.4% belonged to Applied Sciences and Technology, making it the largest group, followed by Social Sciences with 33.9%. In terms of study year, the highest representation was from the fourth year, at 38.4%. Looking at living places, 65.4% resided in the Mess, 18.4% in University Hall, and 16.2% at home. Family income showed a diverse range, with 37.2% falling in the 20,000–40,000 Tk bracket and 38.9% in the 40,001–80,000 Tk range. The majority 76.4% reported no previous experience with dengue, while 23.6% had encountered it before either personally or in their family (Table 1).

**Table 1:** Sociodemographic characteristics of study participants.

Variable	No. (%)
Sex	
Male	261 (62.3)
Female	158 (37.7)
School	
Agriculture and mineral sciences	10 (2.4)
Applied sciences and technology	140 (33.4)
Life sciences	20 (4.8)
Management and business administration	17 (4.1)
Physical sciences	90 (21.5)
Social sciences	142 (33.9)
Study year	
First year	70 (16.7)
Second year	92 (22)
Third year	96 (22.9)
Fourth year	161 (38.4)
Living place	
University hall	77 (18.4)
Mess	128 (65.4)
Home	68 (16.2)
Family income	
Less than 20,000 Tk	16 (3.8)
20,000–40,000 Tk	156 (37.2)
40,001–80,000 Tk	163 (38.9)
More than 80,000 Tk	84 (20.1)
Have you or a family member had dengue before?	
No	320 (76.4)
Yes	99 (23.6)

### 3.2 Overview of Participants' Dengue‐Related Knowledge, Attitudes, and Practices

The study findings indicate that the majority of participants demonstrated good knowledge (68.3%), positive attitudes (85.9%), and good practices (75.9%) regarding dengue. A smaller proportion had poor knowledge (26%) or excellent knowledge (5.7%). Similarly, while most participants exhibited good attitudes, 8.6% had poor attitudes, and 5.5% had excellent attitudes. In terms of practices, 6% had poor practices, whereas 18.1% demonstrated excellent practices (Table 2).

**Table 2:** Overall knowledge, attitudes, and practices regarding dengue fever among university students in the SUST, Bangladesh.

Variables	Frequency	Percent	Mean ± SD
Knowledge score			8.29 ± 1.41
Poor	109	26	
Good	286	68.3	
Excellent	24	5.7	
Attitude score			51.89 ± 4.81
Poor	36	8.6	
Good	360	85.9	
Excellent	23	5.5	
Practice score			6.37 ± 1.24
Poor	25	6	
Good	318	75.9	
Excellent	76	18.1	

### 3.3 Knowledge Regarding Dengue Fever Signs and Symptoms

When asked about their knowledge of dengue fever signs, breeding places, and treatments, most participants knew that fever (98.8%), joint pain (94.3%), and headache (84%) are signs of dengue. In contrast, less than 50% of students know about stomach pain (19.3%), swollen glands (33.9%), painful backbone (42.7%), chills (42.7%), common cold and cough (47%), and skin rash (47.7%). In terms of breeding places, many recognized discarded food containers (87.1%) and flowerpots (87.6%). However, less than fewer knew about tree branches (36.5%) and flowing water (21.2%). Regarding treatment, a large number mentioned plenty of rest (94%) and drinking water abundantly (87.1%). Not many were aware of specific medications like paracetamol and anti‐allergic medicine (58.7%) or traditional herbal remedies (30.5%) (Table 3).

**Table 3:** Knowledge about dengue fever signs and symptoms among university students in the SUST, Bangladesh.

Knowledge items	Correct response
Dengue fever signs and symptoms	
Fever	414 (98.8)
Joint pain	395 (94.3)
Headache	352 (84.0)
Restlessness	310 (74.0)
Muscle pain	290 (69.2)
Nausea and vomiting	287 (68.5)
Pain behind eyes	252 (60.1)
Skin rash	200 (47.7)
Common cold and cough	197 (47.0)
Chills	179 (42.7)
Painful backbone	179 (42.7)
Swollen glands	142 (33.9)
Stomach pain	81 (19.3)
Breeding place	
Flowerpot	367 (87.6)
Discarded food container	365 (87.1)
Unclosed water reservoir	352 (84.0)
Discarded tire	344 (82.1)
Puddle (a small pool of liquid)	308 (73.5)
Open pool of water	208 (49.6)
Tree branch	153 (36.5)
Flowing water	89 (21.2)
Treatment for dengue	
Plenty of rest	394 (94.0)
Drinking water abundantly	365 (87.1)
Taking paracetamol and anti‐allergy medicine	246 (58.7)
Traditional herbal remedies	128 (30.5)

**Figure 3:**
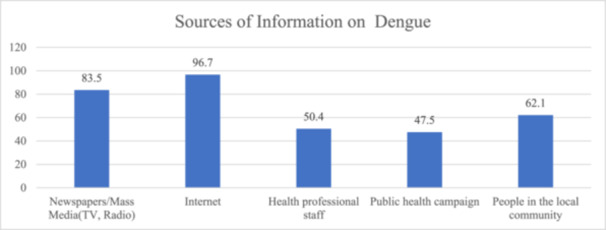
Sources of dengue‐related information among participants.

Figure 3 shows that a significant majority of respondents, 83.5%, rely on newspapers/mass media (TV and radio) as a source of information on dengue. Likewise, 96.7% of respondents use the internet, underscoring the growing significance of online platforms in disseminating information about dengue. Notably, health professionals also play a crucial role, as half of the respondents (50.4%) turn to them for information. Public health campaigns are a source for 47.5% of respondents, highlighting the impact of organized awareness initiatives. Interestingly, people in the local community are a significant source for 62.1% of respondents, emphasizing the role of community‐based knowledge sharing.

### 3.4 Attitudes Toward Dengue Fever

Table 4 displays the attitudes of the study participants regarding DF. Most participants, comprising more than 65% of the respondents, either strongly agreed or agreed that they should be proactive in cleaning Aedes mosquito breeding sites. Over 67% of participants expressed the belief that relying solely on chemical fogging is inadequate for controlling dengue fever. A substantial majority, surpassing 64%, agreed that regularly monitoring the dengue fever situation and promptly seeking medical checkups for family members showing symptoms of DF are crucial. On the other hand, participants also agreed (over 61%) that they share responsibility for maintaining Aedes mosquito‐free housing areas.

**Table 4:** Attitudes toward dengue fever among university students at SUST, Bangladesh.

Statements	SA (*n* (%))*	A (*n* (%))*	N (*n* (%))*	NA (*n* (%))*	SNA (*n* (%))*
Dengue is a serious illness.	145 (34.6)	103 (24.6)	155 (37.0)	8 (1.9)	8 (1.9)
Everyone is at risk of getting dengue fever.	129 (30.8)	109 (26.0)	142 (33.9)	28 (6.7)	11 (2.6)
Children are especially susceptible to dengue.	137 (32.7)	105 (25.1)	141 (33.7)	25 (6.0)	11 (2.6)
Dengue can be repeated (multiple infections)	145 (34.6)	179 (42.7)	95 (22.7)	0 (0%)	0 (0%)
Early stages of dengue are fully treatable.	134 (32.0)	123 (29.4)	128 (30.5)	19 (4.5)	15 (3.6)
Dengue patient needs immediate treatment and hospitalization.	134 (32.0)	107 (25.5)	155 (37.0)	16 (3.8)	7 (1.7)
If you had any signs of dengue, would you see a doctor?	138 (32.9)	102 (24.3)	136 (32.5)	31 (7.4)	12 (2.9)
Are you afraid or have a fear of visiting hospitals?	14 (3.3)	61 (14.6)	147 (35.1)	125 (29.8)	72 (17.2)
Are you concerned about the possibility of coming into contact with individuals exhibiting symptoms of dengue, such as cough, runny nose, sneezing, and fever?	63 (15.0)	87 (20.8)	143 (34.1)	78 (18.6)	48 (11.5)
Is controlling the breeding places of mosquitoes a good strategy to prevent dengue fever?	142 (33.9)	114 (27.2)	133 (31.7)	26 (6.2)	4 (1.0)
Only fogging is enough to control the mosquito population.	8 (1.9)	55 (13.1)	118 (28.2)	165 (39.4)	73 (17.4)
Sleeping in a mosquito/bed net will prevent mosquito bites and dengue infection.	131 (31.3)	112 (26.7)	141 (33.7)	30 (7.2)	5 (1.2)
You will allow health inspectors to conduct inspections for larval breeding sources inside/outside the house.	109 (26.0)	110 (26.3)	154 (36.8)	28 (6.7)	18 (4.3)
Do you agree that the government has taken sufficient preventive measures to prevent the spread of dengue?	6 (1.4)	49 (11.7)	132 (31.5)	155 (37.0)	77 (18.4)
Do you agree that dengue will finally be successfully controlled?	52 (12.4)	107 (25.5)	134 (32.0)	98 (23.4)	28 (6.7)

* SA, strongly agree; A, agree; N, neutral; NA, not agree; SNA, strongly not agree.

### 3.5 Practices for Preventing Dengue Fever

Table 5 shows that a significant percentage of respondents (> 80%) engaged in dengue fever prevention measures. On the positive side, several good practices were commonly reported: over 80% of respondents reported cleaning containers that could collect water (90.7%), using mosquito repellents (79.7%), seeking medical attention when experiencing dengue symptoms (74.7%), and considering mosquito eradication a shared responsibility (89.3%). A large majority (88.5%) were also open to allowing authorities to conduct prevention activities at their residences, and 85.7% encouraged friends and family to adopt preventive measures.

**Table 5:** Preventive practices for dengue fever among university students at SUST, Bangladesh.

Statements	Yes (*n* (%))	No (*n* (%))
Do you clean containers that can collect water (e.g., flowerpots, buckets, and tires) to eliminate potential breeding sites for mosquitoes?	380 (90.7)	39 (9.3)
Do you utilize aerosol and/or liquid mosquito repellent, mosquito coils, electrical mosquito mats, or mosquito bed nets?	334 (79.7)	85 (20.3)
Do you seek medical attention, including tests and treatment, when you observe symptoms of Dengue?	313(74.7)	106 (25.3)
Have you ever checked your residence and property for a container/place that could provide a mosquito breeding site?	238 (56.8)	181 (43.2)
Does your residence have window nets/screens?	140 (33.4)	279 (66.6)
Do you consider eradicating mosquitoes is a shared responsibility?	374 (89.3)	45 (10.7)
Would you allow the authorities to conduct dengue prevention activities at your residence?	371 (88.5)	48 (11.5)
Always wear long‐sleeved clothing if forced out of the house at dusk.	110 (26.3)	309 (73.7)
Do you encourage your friends and family to practice dengue fever prevention measures?	359 (85.7)	60 (14.3)
Have you ever participated in any dengue fever prevention campaigns or initiatives organized by your university or other organizations?	48 (11.5)	371 (88.5)

However, several important preventive practices were poorly followed. Only 56.8% had ever checked their residence for potential mosquito breeding sites, and fewer than one‐third (33.4%) reported having window nets/screens. Wearing long‐sleeved clothing outdoors at dusk was particularly low at 26.3%, and participation in dengue prevention campaigns was minimal (11.5%).

### 3.6 Association in the KAP Domain

The relationships between knowledge, attitudes, and practices among university students at SUST are shown in Table 6. There was no strong correlation between knowledge and attitudes (*r *= 0.025, *p* value = 0.61). Similarly, the connection between knowledge and practices did not indicate a clear relationship (*r* = 0.053, *p* value = 0.28). However, there was a connection between attitudes and practices, although it was not very strong (*r* = −0.092, *p* value = 0.06).

**Table 6:** Spearman correlations assessing the relationships between knowledge, attitudes, and practices among university students at SUST, Bangladesh.

Variable(s)	*r*	*p* value
Knowledge, attitudes	0.025	0.61
Knowledge, practices	0.053	0.28
Attitudes, practices	−0.092	0.06

## 4 Discussion

Given Bangladesh's location in Southeast Asia, vector‐borne illnesses like dengue are seen to pose a serious threat to public health [11]. Numerous worldwide environmental systems are starting to suffer from population increase and the combined effects of emissions and consumption, which eventually prompts diseases like dengue fever [39] to arise. Therefore, it is crucial to exercise utmost vigilance in order to protect against viruses such as Dengue. This study delved into the awareness, attitudes, and practices related to Dengue fever among students at SUST in Bangladesh to assess the ability to deal with this contemporary situation.

Our findings offer valuable insights into the knowledge level, attitudes, and preventive practices of the participants. The participants demonstrated varying levels of awareness regarding dengue fever signs, breeding places, and treatment. Most were aware of common symptoms like fever, joint pain, and headache, reflecting a solid understanding of the disease. Similar studies support our result, where the majority of respondents point out that high fever, joint pain, headache, and backache are the most typical dengue symptoms [40, 41, 42]. Even though students generally knew a lot about dengue, many did not recognize important warning signs like stomach pain (19.3%), chills (42.7), and skin rash (47.7%). This gap means they might not take these symptoms seriously or seek medical help quickly, which could put them at greater risk of severe illness.

Allocating breeding places is extremely important to hinder the proliferation of Aedes mosquitoes. In our research, recognition of breeding places such as discarded food containers and flowerpots was substantial among the participants. Our findings are completely supported by a few additional studies that go even beyond, where they show how different types of containers play a significant role as breeding places for mosquitoes, and water from these containers must be removed to prevent dengue infection [43, 44, 45]. However, there were gaps in knowledge about less obvious breeding sites like tree branches and flowing water.

Intravenous (IV) fluid rehydration, paracetamol, anti‐bacterial, and anti‐viral are good initiatives for suspected dengue, according to the public health sector health‐care professionals [46]. Regarding treatment, we discovered that a considerable number acknowledged the importance of rest and hydration, and 58.7% of participants correctly identified “Taking paracetamol and anti‐allergic medicine” prescribed by doctors as a treatment for dengue.

The significance of newspapers and mass media (83.5%), as well as the internet (96.7%), in raising awareness has been repeatedly demonstrated by research. It's worth noting that people are increasingly using social media and avoiding traditional media like radio and television. A study claims that social media platforms like Twitter are essential for dengue prevention and monitoring [47]. The media and the internet's role in educating and reminding the public about dengue was shown to be positively trending, and these studies also suggest that dengue received significantly more digital media coverage than influenza and malaria [45, 48]. While this reflects widespread access to health‐related content, it is important to acknowledge that such platforms do not always provide accurate or evidence‐based information [49]. Therefore, information obtained from these sources should be verified through credible and authoritative channels, such as public health institutions and qualified healthcare professionals.

Health professionals were a crucial source for half of the participants (50.4%), emphasizing the role of healthcare providers in disseminating information. Public health campaigns and local communities also contributed significantly to the participants' knowledge, with percentages of 47.5% and 62.1%, respectively. These findings corroborate earlier research showing that public health practitioners had extensive knowledge about dengue fever and can therefore serve as a valuable source [46]. Different communities can also help to improve awareness to prevent dengue [50].

The attitudes of the participants towards Dengue fever were generally positive. The majority agreed on the severity of Dengue as an illness and the risk it poses to everyone. According to a different survey, dengue was regarded as a “serious problem” by about 89% of study participants [51]. Participants recognized the vulnerability of children to Dengue and acknowledged the possibility of multiple [52, 53]. However, the large percentage of “Neutral” answers on numerous important statements (such as dengue severity, universal danger, and the necessity of emergency hospitalization) warrants significant attention, even though the overall attitude score seems to be good. This pattern of behavior points to some hesitancy or lack of commitment, which could have a detrimental effect on real preventive actions. Even while the total attitude score appears to be positive on paper, a high percentage of neutral responses to basic beliefs is problematic from the standpoints of behavioral science and public health.

The study revealed that a substantial percentage of participants actively engaged in preventive practices against Dengue. Cleaning water‐collecting containers, using mosquito repellents, seeking medical treatment for Dengue symptoms, and considering mosquito control a shared responsibility were common practices. Furthermore, using a fan in the house, mosquitoes' coil, covering water containers, cleaning up the surroundings area, proper disposal of household garbage, proper disposal of items that can collect rainwater, etc., seem to be fundamental practices shown in other research [33, 50]. Nonetheless, certain behaviors that need greater attention, like actively searching for breeding grounds and taking part in preventative initiatives, were less common in our data. Encouragingly, a high percentage of participants advocated Dengue prevention measures to friends and family. Students claimed to follow dengue preventative measures however, several daily routines were still lacking. They seldom participated in dengue prevention initiatives, hardly ever wore long sleeves, and frequently lacked window screens. These gaps reduce the effectiveness of house protection and increase their vulnerability to mosquito bites, particularly during peak insect activity. Lack of participation in community prevention initiatives also makes it more difficult to manage Aedes mosquitoes in the surrounding environment, raising everyone's risk of contracting dengue.

The analysis of the relationships between knowledge, attitudes, and practices yielded interesting insights. While there was no strong link between knowledge and attitudes or knowledge and practices, there was a hint of a connection between attitudes and practices. These results suggest that knowing about dengue may not directly change how people act or think, but it could indirectly affect how they feel about it, and that might influence what they do [54, 55]. Knowing facts does not always result in changing one's conduct. People may comprehend health facts, but for reasons of habit, convenience, perceived danger, or lack of drive, they may still choose not to follow advised behaviors. Other research extensively documented this “knowledge behavior gap” [56, 57]. Furthermore, societal norms, cultural beliefs, familial influence, and community expectations frequently have a greater influence on attitudes and behaviors than information alone. Behavior may adhere to societal standards rather than personal comprehension in environments with strong collective views. Even with sufficient information, people might not have the tools, access, or supportive environment needed to put that knowledge into practice. This critical thinking is also supported by previous studies, which show that greater information does not always translate into better dengue measures [58]. Although a study suggests there was a significant (*p* < 0.05) correlation between knowledge and preventive practices, the degree of preventive practice was somewhat lower than the level of knowledge [40].

## 5 Strengths and Limitations of This Study

Our research offers valuable insights into the dengue‐related perceptions of university students, laying the groundwork for more extensive and diverse studies in the future. Our study's conclusions will be used as a reference by health care planners to improve the dengue management strategy. KAP research will offer an appropriate framework for assessing current initiatives and determining successful tactics for altering behavior [59].

While our study provides valuable insights into the knowledge, attitudes, and practices regarding Dengue Fever among university students, it is essential to acknowledge certain limitations. Firstly, the cross‐sectional nature of the study design restricts our ability to establish causal relationships between variables. Additionally, the reliance on self‐reported data may introduce response bias, as participants might provide socially desirable answers rather than reflecting their actual behaviors. The study's focus on a specific demographic of university students from SUST limits the generalizability of findings to broader populations.

## 6 Conclusions

Though the majority of survey participants were familiar with the facts regarding dengue disease, including its route of transmission, signs and symptoms, and treatment options, knowledge had the lower mean score. Mitigating DF risk requires a combination of knowledge, attitudes, and practices; however, there is a negative correlation between attitudes and practices. The results emphasize the need for efficient information sharing, community encouragement for a positive attitude, and consistent observation of preventative measures to guarantee DF control. In order to control DF, improving knowledge among university students is a must, according to our findings. There was a very small correlation between knowledge and attitude, knowledge and practice, emphasizing the need to increase one's sense of responsibility towards society to have a significant preventive effect on DF. The findings can assist health authorities in estimating the KAP level of university students, which can then be taken into account when developing future training programs.

## 7 Recommendations

Universities should concentrate on making dengue information relevant and useful for students rather than just disseminating it. They must stress when immediate medical attention is required and provide a clear explanation of the main warning signals, which include severe stomach discomfort, chills, and skin rash. Students can develop easy yet efficient habits by wearing long sleeves during mosquito peak hours, using window screens, and encouraging frequent cleaning of the campus and dorms. Above all, initiatives ought to be student‐led and interactive. Universities may transform knowledge into real concern and persistent preventive action by making involvement visible and rewarding, and by substituting peer‐driven activities for passive messages. Programs including physical activity won't be successful unless they focus on increasing awareness and altering understanding. Providing up‐to‐date information about dengue is essential, as diseases often change their signs and symptoms over time. Short videos and early alerts on DF epidemic containment techniques may be available on television and social media. Basic dengue fever treatment training should be included in the government and non‐government campaigns. Encouraging greater adoption of proactive measures, such as routinely checking for breeding sites, installing window nets or screens, wearing protective clothing, and increasing population participation in prevention campaigns, is essential to enhancing dengue fever prevention efforts.

## Author Contributions

**Md. Fakrul Islam:** writing – original draft, visualization, validation, software, methodology, data curation, formal analysis, methodology, project administration, and conceptualization. **Md. Efty Islam Arpon:** data curation, writing – original draft, writing – review and editing. **Md. Nazmul Alam:** data curation, writing – original draft, writing – review and editing. **Md. Abu Tawab Hridoy:** data curation, writing – original draft, writing – review and editing. **Fahmida Afroze:** data curation, writing – original draft, writing – review and editing. **Md. Mazharul Islam:** data curation, writing – review and editing. **Md Azizul Baten:** project administration, writing – review and editing, visualization, resources, validation, supervision, methodology, investigation, and conceptualization.

## Funding

The authors received no specific funding for this work.

## Ethics Statement

This study received ethical approval from the Ethical Review Board of Shahjalal University of Science and Technology. The written consents were obtained from the study participants before collecting data, mentioning that all the collected data would be confidential. Data will not be shared outside of this research.

## Conflicts of Interest

The authors declare no conflicts of interest.

## Transparency Statement

The corresponding author, Md. Azizul Baten affirms that this manuscript is an honest, accurate, and transparent account of the study being reported; that no important aspects of the study have been omitted; and that any discrepancies from the study as planned (and, if relevant, registered) have been explained.

## Supporting information

Questionnaire.

STROBE checklist.

## Data Availability

The data that support the findings of this study are available on request from the corresponding author. The data are not publicly available due to privacy or ethical restrictions.
