# Supplementary material for: Unveiling the Dengue Knowledge Gap and Symptom Awareness Among Students of Shahjalal University of Science and Technology, Sylhet: A Cross‐Sectional Study
Source: Health Sci Rep. 2026 Mar 26;9(4):e72208. doi: 10.1002/hsr2.72208 (PMC13087640; doi:10.1002/hsr2.72208)
Supplement: Supplementary file 1 — Questionnaire. [file HSR2-9-e72208-s001.docx]

This questionnaire is prepared for collecting data to conduct a research work on “**Knowledge, Attitude and Preventive Practices towards Dengue Outbreak among Students of SUST, Sylhet”** by students of the Statistics 28^th^ batch, SUST. This online/offline survey will take 4-5 minutes. Your Contribution will be highly appreciated. Please read the questions very well and provide your answers accordingly. Please note that all the information you provide us is totally **CONFIDENTIAL** and would be used for the purposes of project work only.

Thanks in advance for your cooperation.

# **Socio-demographic Information**

| Student’s Registration ID |  |
| --- | --- |
| Study Year |  |
| Department |  |
| Contact no |  |
| Age |  |

1. Gender

- Male
- Female
- Others

1. Where do you live in Sylhet?

- University Hall
- Mess
- Home

1. What is the approximate total monthly income of your family?

- Less than 20,000 Tk
- 20,000 to 40,000 Tk
- 40,001 to 80,000 Tk
- 80,001 to 100,000 Tk
- More than 100,000 Tk

1. What factors influence your decision to adopt or not adopt dengue prevention practices?

- Knowledge
- Convenience
- Cost
- Peer Pressure

1. Have you had Dengue before or your family member?

- Yes
- No

If yes, what kind of preventive measures taken at a time?

…………………………………………………………………………………………………

**PART A: KNOWLEDGE ON DENGUE, DENGUE SPREAD, VECTOR AND SYMPTOMS**

**SYMPTOMS**

| **No.** | **Questions** | **Yes** | **No** |
| --- | --- | --- | --- |
| 1 | Is Dengue an infectious disease? |  |  |
| 2 | Are you aware of Dengue? |  |  |
| 3 | Do you know Dengue fever can cause death? |  |  |
| 4 | Is the Dengue virus transmitted only by female Aedes mosquitoes? |  |  |
| 5 | Do you know Dengue virus can be transmitted from infected pregnant mother to fetus? |  |  |
| 6 | Dengue is transmitted to human by bites of infective mosquitoes |  |  |
| 7 | Humans get infected by dengue by drinking dirty water |  |  |
| 8 | Are there specific treatments for dengue? |  |  |
| 9 | Mosquitoes that transmit dengue virus bite only during day |  |  |
| 10 | The mosquitoes that transmit dengue virus lay their eggs in dirty water |  |  |
| 11 | Only way to prevent dengue is by eliminating breeding grounds of dengue |  |  |
| 12 | Do you think Dengue infection can be reduced by keeping your surrounding areas clean and destroying potential breeding sites? |  |  |
| 13 | Is the rainy season when dengue cases most frequently occur? |  |  |

**14. What one or more type of mosquito carries dengue?**

| **No.** | **Type of mosquito** | **Yes** | **No** |
| --- | --- | --- | --- |
| 1 | *Culex* |  |  |
| 2 | *Aedes* |  |  |
| 3 | *Anopheles* |  |  |
| 4 | *Mansonia* |  |  |

**15. What are the signs of Dengue?**

| **No.** | **Sign** | **Yes** | **No** |
| --- | --- | --- | --- |
| 1 | Fever |  |  |
| 2 | Chills |  |  |
| 3 | Nausea and vomiting |  |  |
| 4 | Headache |  |  |
| 5 | Joint pain |  |  |
| 6 | Muscle pain |  |  |
| 7 | Pain behind eyes |  |  |
| 8 | Painful backbone |  |  |
| 9 | Stomach pain |  |  |
| 10 | Bleeding of nose, gums and skin |  |  |
| 11 | Skin rash |  |  |
| 12 | Common cold and Cough |  |  |
| 13 | Swollen glands |  |  |
| 14 | Restlessness |  |  |

**16. At what time of the day are dengue carriers more likely to cause infection in humans?**

| **No.** | **Blood sucking time** | **Yes** | **No** |
| --- | --- | --- | --- |
| 1 | Dawn and late afternoon |  |  |
| 2 | Morning and noon |  |  |
| 3 | Evening and midnight |  |  |
| 4 | All the time |  |  |

**17. Which one or more of the following is a breeding place for dengue carriers?**

| **No.** | **Breeding place** | **Yes** | **No** |
| --- | --- | --- | --- |
| 1 | Discarded food container |  |  |
| 2 | Discarded tyre |  |  |
| 3 | Tree branch |  |  |
| 4 | Unclosed water reservoir |  |  |
| 5 | Flowerpot |  |  |
| 6 | Open pool of water |  |  |
| 7 | Flowing water |  |  |
| 8 | Puddle (a small pool of liquid) |  |  |

**18. Which one or more of the following is a treatment for dengue?**

| **No.** | **Treatment** | **Yes** | **No** |
| --- | --- | --- | --- |
| 1 | Plenty of rest |  |  |
| 2 | Drinking water abundantly |  |  |
| 3 | Taking panadol (paracetamol) & Fexo Fenadine |  |  |
| 4 | Traditional herbal remedies |  |  |

**19**. **How do you receive information on dengue?**

| **No** | **Source of information** | **Yes** | **No** |
| --- | --- | --- | --- |
| 1 | Books/newspapers/pamphlets |  |  |
| 2 | Media mass (TV, Radio) |  |  |
| 3 | Internet |  |  |
| 4 | Health profession staff |  |  |
| 5 | Public health campaign |  |  |
| 6 | People in the local community |  |  |

**PART B: ATTITUDE TOWARDS PREVENTION OF DENGUE FEVER**

| **No** | **Questions** | **Strongly Agree Disagree** | **Agree** | **Neutral** | **Not Agree** | **Strongly Not Agree** |
| --- | --- | --- | --- | --- | --- | --- |
| 1 | Dengue is a serious illness |  |  |  |  |  |
| 2 | Everyone is at risk of getting dengue fever |  |  |  |  |  |
| 3 | Children are especially susceptible to dengue |  |  |  |  |  |
| 4 | Dengue can be repeated (multiple infections) |  |  |  |  |  |
| 5 | Early stages of dengue are fully treatable |  |  |  |  |  |
| 6 | Dengue patient needs immediate treatment and hospitalization |  |  |  |  |  |
| 7 | If you had any signs of dengue, would you see a doctor? |  |  |  |  |  |
| 8 | Are you afraid of fear of visiting hospitals |  |  |  |  |  |
| 9 | Are you afraid of fear of being in contact with people with dengue symptoms (e.g., cough, runny nose, sneezing, fever) |  |  |  |  |  |
| 10 | Is controlling the breeding places of mosquitoes a good strategy to prevent dengue fever? |  |  |  |  |  |
| 11 | Only fogging is enough to control mosquito population |  |  |  |  |  |
| 12 | Sleeping in mosquito/bed net will prevent mosquito bites and dengue infection |  |  |  |  |  |
| 13 | You will allow health inspectors to conduct inspections for larval breeding sources inside/outside house |  |  |  |  |  |
| 14 | Do you agree that government has taken sufficient preventive measures to prevent the spread of dengue? |  |  |  |  |  |
| 15 | Do you agree that dengue will finally be successfully controlled? |  |  |  |  |  |

**PART C: PRACTICES AGAINST DENGUE INFECTION**

| **No.** | **Question** | **Yes** | **No** |
| --- | --- | --- | --- |
| 1 | Do you clean containers that can collect water (e.g., flowerpots, buckets, and tires) to eliminate potential breeding sites for mosquitoes? |  |  |
| 2 | Do you Use aerosol and/or liquid mosquito repellent and/or mosquito coil and/or electrical mosquito mat and/or mosquito bed net? |  |  |
| 3 | Do you go to hospital for tests and treatment when you see the symptoms of Dengue? |  |  |
| 4 | Have you ever checked your residence and property for a container/place that could provide a mosquito breeding site? |  |  |
| 5 | Does your residence have window nets/screens? |  |  |
| 6 | Do you consider eradicating mosquitoes is a shared responsibility? |  |  |
| 7 | Would you allow the authorities to conduct dengue prevention activities at your residence? |  |  |
| 8 | Do always wear long-sleeved clothing if forced out of the house at dusk. |  |  |
| 9 | Do you encourage your friends and family to practice dengue fever prevention measures? |  |  |
| 10 | Have you ever participated in any dengue fever prevention campaigns or initiatives organized by your university or other organizations? |  |  |
